# Urgent Colectomy for Colonic Necrosis Following Shiga Toxin-Producing *Escherichia coli* O157 Infection in an Adult

**DOI:** 10.70352/scrj.cr.26-0457

**Published:** 2026-10-16

**Authors:** Kenta Baba, Tatsuaki Sumiyoshi, Takeshi Kobayashi, Yoshifumi Kondo, Sho Tazuma, Keisuke Okano, Takeshi Muto, Naokuni Tatsumoto, Shinya Takahashi

**Affiliations:** 1 Department of Surgery, NHO Kure Medical Center and Chugoku Cancer Center, Kure, Hiroshima, Japan; 2 Department of Surgery, Graduate School of Biomedical and Health Science, Hiroshima University, Hiroshima, Hiroshima, Japan; 3 Department of Surgery and Pediatric Surgery, Hiroshima City Funairi Citizens Hospital, Hiroshima, Hiroshima, Japan; 4 Department of Surgery, JA Hiroshima General Hospital, Hatsukaichi, Hiroshima, Japan; 5 Department of Surgery, Miyoshi Central Hospital, Hiroshima, Miyoshi, Japan; 6 Muto Internal Medicine, Surgery and Gastroenterology Clinic, Hiroshima, Hiroshima, Japan

**Keywords:** *Escherichia coli* O157, enterohemorrhagic *Escherichia coli*, colonic necrosis, right hemicolectomy, hemolytic uremic syndrome

## Abstract

**INTRODUCTION:**

Shiga toxin-producing *Escherichia coli* (STEC) O157 infection is usually treated conservatively; surgery is rarely required and mostly reserved for children with hemolytic uremic syndrome (HUS). We report a case of STEC O157 requiring urgent surgery in an adult without HUS.

**CASE PRESENTATION:**

A 73-year-old woman presented with abdominal pain and diarrhea. Abdominal CT showed diffuse wall thickening in the ascending and transverse colon. Infectious enteritis and acute exacerbation of chronic renal failure were diagnosed, and antibiotics and continuous hemodiafiltration were initiated. On day 5 of hospitalization, necrotic ischemic colitis was suspected owing to bloody diarrhea and contrast-enhanced CT findings of wall thinning of the ascending and transverse colon. On laparotomy, the ascending colon and proximal transverse colon were necrotic, and urgent surgery (right hemicolectomy) was performed. Postoperatively, the stool sample provided at admission was found to be positive for *Escherichia coli* O157 and Shiga toxin.

**CONCLUSIONS:**

The postoperative course was uneventful, and dialysis was discontinued. This case highlights that STEC O157 infection can rapidly progress to life-threatening colonic necrosis in adults without HUS, requiring timely surgical intervention.

## Abbreviations

Gb3
: globotriaosylceramide
HUS
: hemolytic uremic syndrome
LDH
: lactate dehydrogenase
STEC
: Shiga toxin-producing *Escherichia coli*
vWF
: von Willebrand factor
WBC
: white blood cell

## INTRODUCTION

STEC O157 often causes gastroenteritis, especially in children.^1,2)^ Most abdominal symptoms are mild, and most patients recover after conservative treatment alone. However, on rare occasions, urgent surgery is required for severe cases with intestinal necrosis or perforation. Urgent surgery is common in pediatric patients with HUS but extremely rare in adult patients.^3,4)^ We report a case of STEC O157 infection requiring urgent surgery in an adult without HUS.

## CASE PRESENTATION

A 73-year-old woman with a history of diabetes and chronic renal failure experienced abdominal pain and diarrhea for 2 days and was referred to Miyoshi Central Hospital, where she underwent treatment and surgery. Although her abdomen was distended, peritoneal irritation signs were absent. Laboratory tests revealed an elevated inflammatory response (WBC count, 26400/µL, C-reactive protein, 20.9 mg/dL), mild anemia (hemoglobin, 11.0 g/dL), and deteriorated renal function (serum creatinine, 12.29 mg/dL). The platelet count was 35.4 × 10^4^/μL, the serum LDH level was 235 U/L, and total bilirubin was 0.2 mg/dL. Abdominal CT showed diffuse wall thickening with submucosal edema in the ascending and transverse colon (**Fig. 1**). She was diagnosed with infectious enteritis and acute exacerbation of chronic renal failure. Intravenous meropenem and oral metronidazole were initiated on admission, and continuous hemodiafiltration was started. On day 2 of hospitalization, a stool sample tested negative for C. difficile toxin; therefore, oral metronidazole was discontinued.

**Fig. 1:**
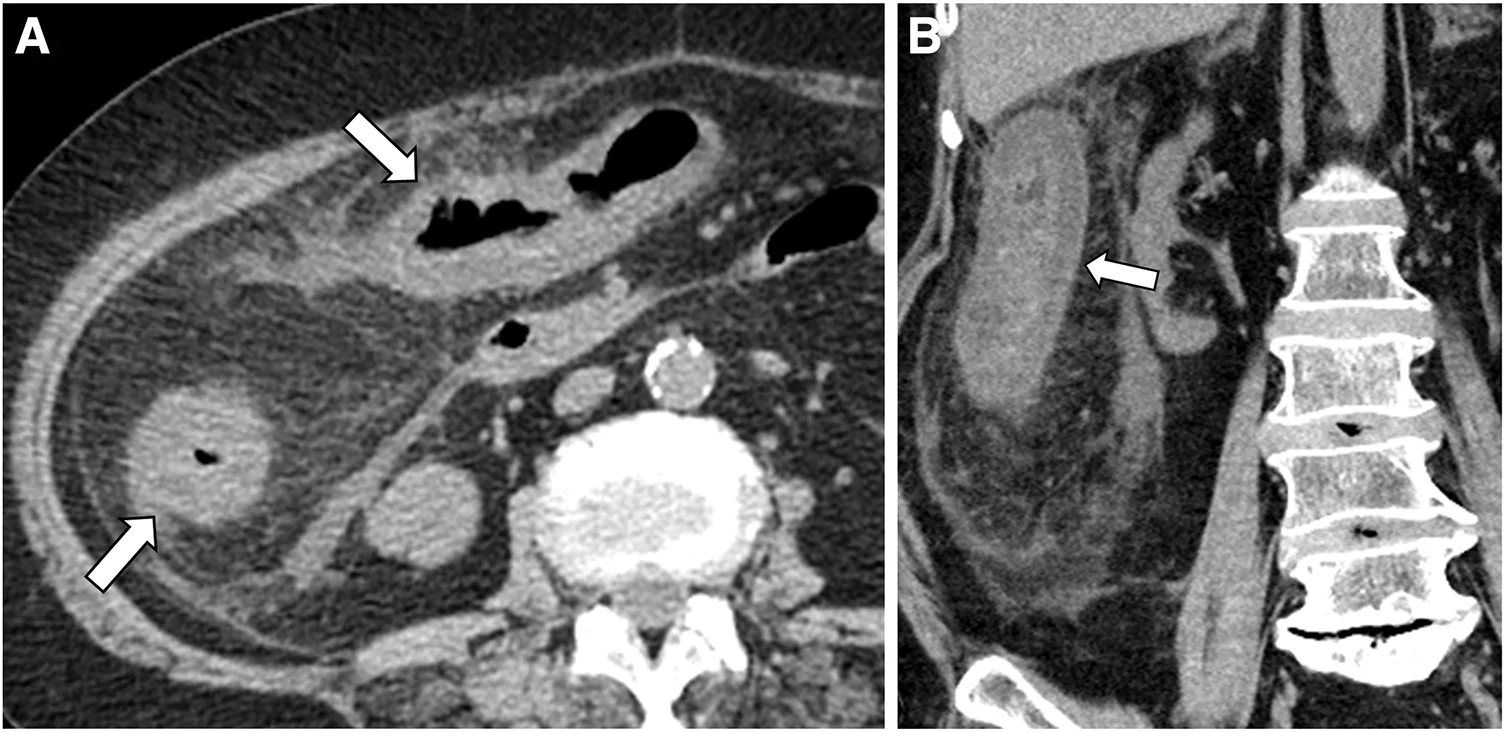
Abdominal CT images showing diffuse wall thickening with submucosal edema in the ascending and transverse colon (arrows). (**A**) Axial image. (**B**) Coronal image.

On day 5 of hospitalization, she had bloody diarrhea, and her WBC count had increased to approximately 40000/µL. Immediately before surgery, her hemoglobin was 8.8 g/dL, the platelet count was 15.7 × 10^4^/μL, the serum LDH level was 229 U/L, and total bilirubin was 0.3 mg/dL; no schistocytes were observed on peripheral blood smear. Contrast-enhanced CT showed marked wall thinning with decreased contrast enhancement of the ascending colon and massive ascites around the right paracolic gutter (**Fig. 2**). The diagnosis was updated to necrotic ischemic colitis. Given the rapid clinical deterioration, marked leukocytosis, and CT findings suggestive of transmural necrosis, emergency surgery was indicated. Laparotomy revealed that the ascending colon and proximal transverse colon were necrotic, and right hemicolectomy with concomitant construction of an ileostomy and mucous fistula in the transverse colon was performed (**Fig. 3**). The resected specimen demonstrated extensive necrosis from the ascending colon to the proximal transverse colon (**Fig. 4**).

**Fig. 2:**
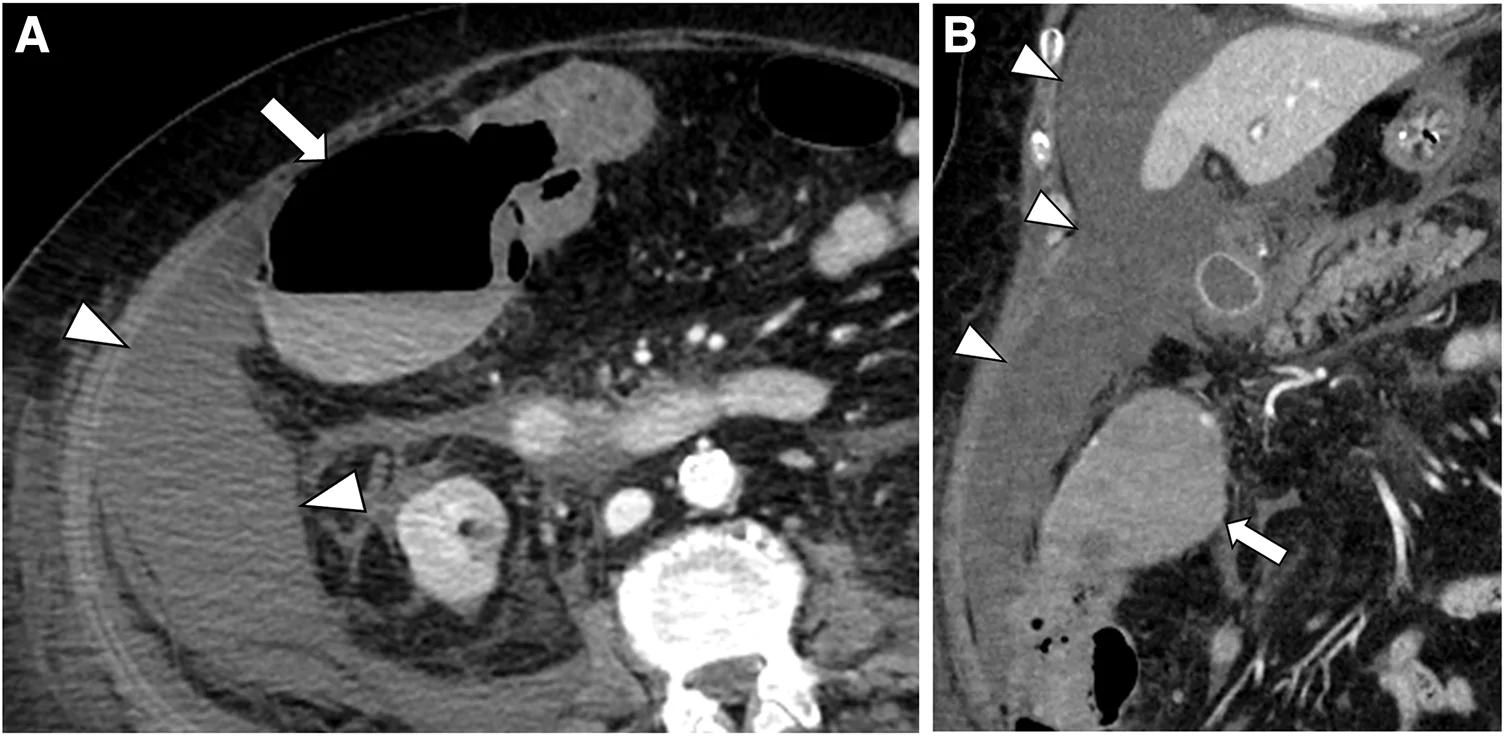
Abdominal contrast-enhanced CT images showing marked wall thinning of the ascending and transverse colon (arrows), and massive ascites around the right paracolic gutter (arrowheads). (**A**) Axial image. (**B**) Coronal image.

**Fig. 3:**
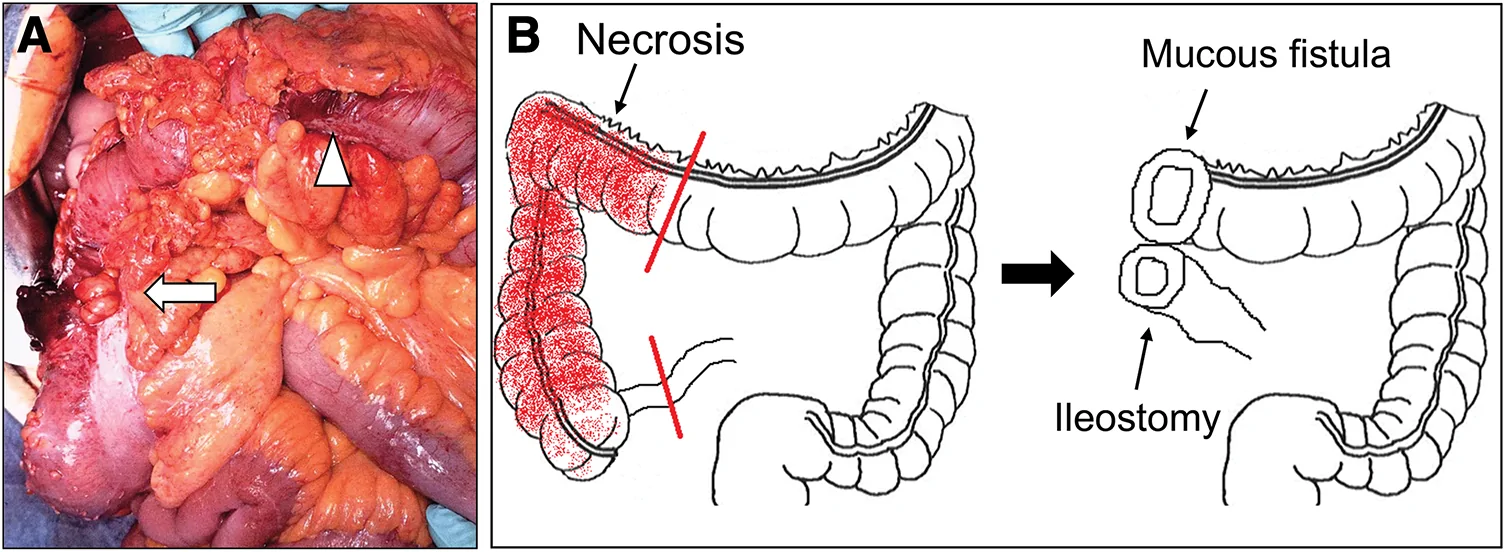
(**A**) Surgical findings. Intestinal necrosis in the ascending colon (arrow) and proximal transverse colon (arrowhead) is seen. (**B**) Schema showing the area of intestinal necrosis and surgical procedure. The red lines indicate the resection line for right hemicolectomy. The surgical procedures performed are right hemicolectomy with ileostomy and construction of a mucous fistula at the transverse colon.

**Fig. 4:**
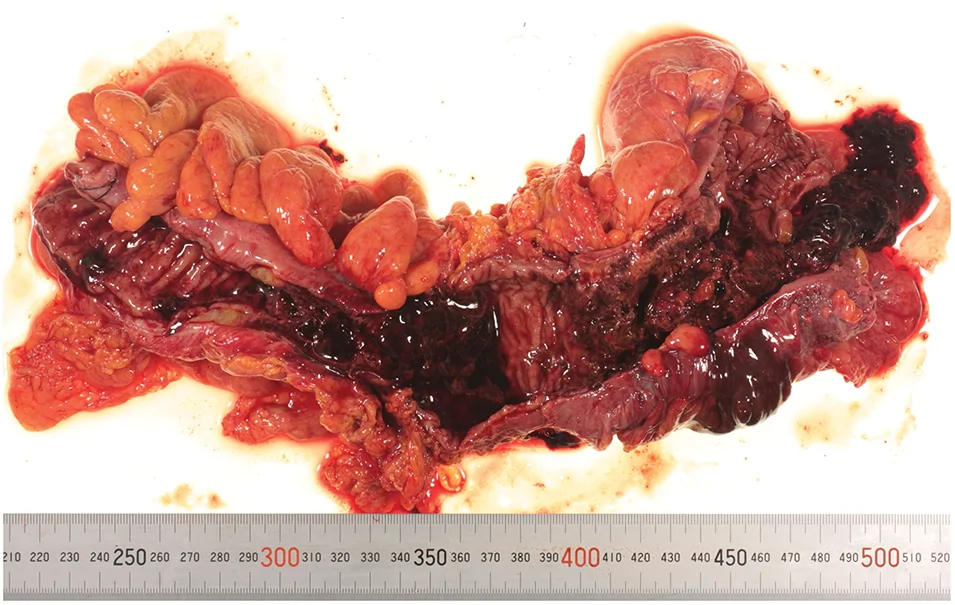
Resected specimen showing extensive necrosis from the ascending colon to the proximal transverse colon.

Histopathologic examination revealed findings consistent with necrotic hemorrhagic colitis, including pseudomembrane formation over the mucosa, mucosal necrosis, and submucosal hemorrhage in the cecum and ascending colon (**Fig. 5A**). Inflammatory changes extended from the deep submucosa into the muscularis propria, and focal transmural inflammatory infiltration was observed in a portion of the cecum. Definite necrosis extending into the muscularis propria or involving the full thickness of the bowel wall could not be confirmed histopathologically. In addition, fibrin thrombi were observed in small vessels. Immunohistochemical staining for vWF demonstrated numerous vWF-positive thrombi in capillaries and small vessels, and phosphotungstic acid hematoxylin staining confirmed fibrin thrombi in small vessels (**Fig. 5B** and **5C**). The stool sample provided on admission was found to be positive for *Escherichia coli* O157 and Shiga toxin on day 5 of hospitalization following surgery; hence, the cause of the colonic necrosis was STEC O157 infection. When interviewed, the patient revealed that she had eaten beef liver 7 days before hospitalization. Her postoperative course was uneventful. Hemodialysis was discontinued, and she was discharged on the 69th POD.

**Fig. 5:**
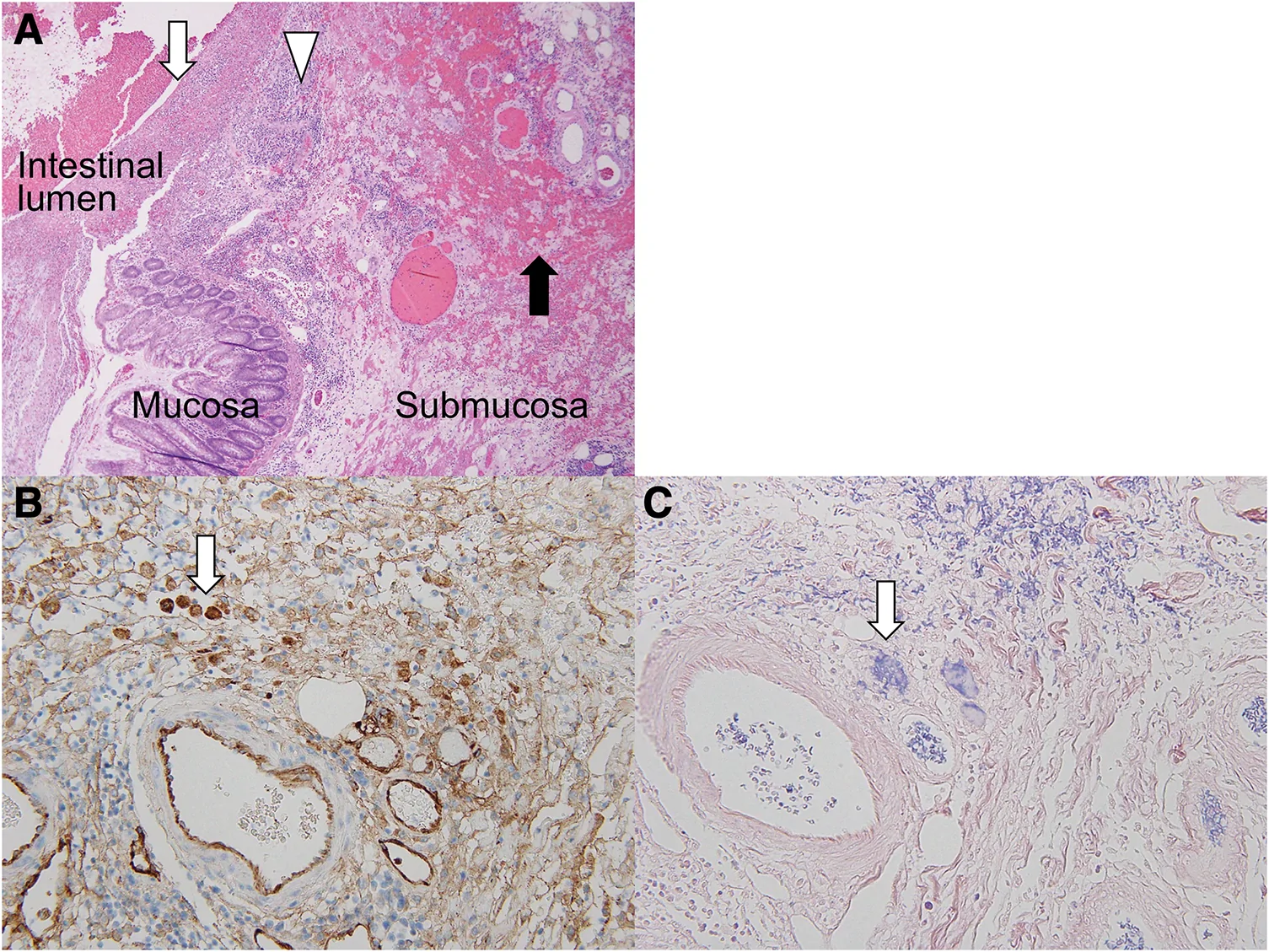
(**A**) Histological findings of the resected colon (hematoxylin eosin stain, original magnification, ×40). Pseudomembrane formation over the mucosa (white arrow), mucosal necrosis (arrowhead), and submucosal hemorrhage (black arrow) are seen. (**B**) Immunohistochemical staining for vWF showing vWF-positive thrombi in capillaries and small vessels (arrow). (**C**) Phosphotungstic acid hematoxylin staining showing fibrin thrombi in small vessels (arrow). vWF, von Willebrand factor

## DISCUSSION

*Escherichia coli* O157 adheres to intestinal epithelial cells and causes hemorrhagic colitis by producing Shiga toxin.^5–7)^ Most STEC O157 infections result from ingestion of undercooked ground beef or cattle manure-contaminated water, vegetables, fruit, or milk.^2,7)^ The STEC O157 infection in this case was presumably caused by beef liver consumed by the patient 7 days before hospitalization.

STEC O157 infection is usually treated conservatively, and surgery is rarely required.^2)^ Most previous urgent enterectomies were performed on pediatric patients, all of whom had HUS.^8–11)^ Therefore, colonic necrosis and perforation following STEC O157 infection have been associated with the development of HUS.^2,12)^

Gb3, the receptor for Shiga toxin, is highly expressed in the vascular endothelial cells of the kidney and brain and the Paneth cells of the intestinal mucosa. Its expression is lower in adults than that in children,^2,13,14)^ as is the incidence of HUS and acute encephalopathy following STEC O157 infection.^15,16)^ Neither Gb3 expression nor HUS incidence is sex-related in pediatric patients, whereas both are higher in adult female patients than in adult male patients. Although the patient in our case was an adult woman without HUS, her colon was necrotic and urgent surgery was required. HUS is characterized by microangiopathic hemolytic anemia, thrombocytopenia, and acute kidney injury. Although renal dysfunction could not be used to assess HUS because of pre-existing chronic renal failure, the platelet count decreased during the clinical course but remained within the normal range, and there was no biochemical or morphological evidence of microangiopathic hemolysis, including elevated LDH or total bilirubin levels or the presence of schistocytes. Therefore, the patient was considered not to have developed HUS.

The patient had several potential risk factors for intestinal ischemia, including advanced age, diabetes, and chronic renal failure. Although no occlusion of the major mesenteric arteries was identified on contrast-enhanced CT, non-occlusive mesenteric ischemia could not be completely excluded. The initial CT demonstrated diffuse edematous wall thickening from the ascending to transverse colon, followed by wall thinning and decreased contrast enhancement in the same region as the patient’s condition deteriorated. Together with the detection of STEC O157 and Shiga toxin in the stool, these sequential findings suggested that severe STEC-associated colitis played a major role in the development of colonic necrosis. Nevertheless, given the patient’s underlying conditions, concomitant ischemic mechanisms may have contributed to the progression of intestinal injury.

PubMed and the Japan Medical Abstracts Society database were searched through August 2026 using English and Japanese terms for STEC O157, intestinal resection, and surgery. Reference lists of relevant articles were also screened, and adult cases requiring intestinal resection were included. To our knowledge, only 15 adult cases of enterectomy following STEC O157 infection have been reported; among them, 6 (40%) included HUS and 10 (67%) involved women (**Table 1**).^3,4,17–28)^ These findings indicate that STEC O157 infection can progress to colonic necrosis or perforation in adults even in the absence of HUS. Although women accounted for 67% of the reported adult cases requiring intestinal resection, this finding should be interpreted with caution because the reported cases represent a highly selected population with severe disease. Selection bias and publication bias, as well as the absence of a comparison group including conservatively treated patients, preclude conclusions regarding sex-related differences in the risk of severe complications. Several previously reported patients had comorbidities that might have influenced intestinal ischemia or disease severity; however, these comorbidities varied considerably among cases (**Table 1**). Given the limited number of reported cases, no conclusions can be drawn regarding the association between specific comorbidities and the development of intestinal necrosis. In the previously reported cases, total or subtotal colectomy was performed in approximately half of the patients, reflecting the extent of colonic involvement. In contrast, necrosis in the present case was confined to the ascending colon and proximal transverse colon, allowing right hemicolectomy with preservation of the remaining colon.

**Table 1:** Characteristics of adults who underwent intestinal resection following STEC O157 infection

No.	Author	Year	Age	Sex	Comorbidities	Onset to surgery (days)	Admission to surgery (days)	Indication	Procedure	HUS	Outcome
1	Tsujimoto^17)^	1998	58	M	HT	2	1	Peritonitis	RHC	−	Alive
2	Seo^18)^	1998	64	F	None	5	0	Peritonitis	ICR	−	Alive
3	Nakagawa^19)^	1998	33	F	None	37	36	Hemorrhagic preshock	SC + ileostomy	+	Alive
4	Hattori^20)^	2000	72	F	None	3	1	Peritonitis	ERHC + ileostomy	+	Dead
5	Fukui^21)^	2000	69	M	Arrhythmia	3	0	Peritonitis	ERHC	−	Alive
6	Tamiya^22)^	2000	72	F	None	3	1	Peritonitis	ERHC + ileostomy	+	Dead
7	Kravitz^3)^	2002	48	F	HT, ET	12	1	Intestinal perforation	TC + ileostomy	−	Alive
8	Hoashi^23)^	2002	61	F	MR	7	3	Peritonitis	RHC + ileostomy	−	Alive
9	Maruyama^24)^	2004	58	F	Systemic sclerosis	4	3	Peritonitis	ERHC + ileostomy	+	Alive
10	Wakiyama^25)^	2006	80	F	HT, PD	3	2	Peritonitis	Ileostomy	−	Alive
11	Kendrick^4)^	2007	59	M	Hypothyroidism	4	2	Peritonitis	TC + diverting ileostomy	−	Alive
12	Tominaga^26)^	2014	81	M	None	3	3	Peritonitis	SC + ileostomy	+	Alive
13	Hamasaki^27)^	2015	42	F	SLE	2	2	Peritonitis	SC + ileostomy + mucous fistula	−	Alive
14	Adachi^28)^	2023	70	M	HT, DM, RA	2	2	Shock	SC + ileostomy	+	Alive
15	Present case	2026	73	F	DM, CKD	7	4	Necrotic colitis	RHC + ileostomy + mucous fistula	−	Alive

CKD, chronic kidney disease; DM, diabetes mellitus; ERHC, extended right hemicolectomy; ET, essential thrombocythemia; F, female; HT, hypertension; HUS, hemolytic uremic syndrome; ICR, ileocecal resection; M, male; MR, mitral regurgitation; PD, Parkinson’s disease; RA, rheumatoid arthritis; RHC, right hemicolectomy; SC, subtotal colectomy; SLE, systemic lupus erythematosus; STEC, Shiga toxin-producing Escherichia coli; TC, total colectomy

In the 15 reported adult cases of STEC O157 infection, the median time from symptom onset to surgery was 3 days and from admission to surgery was 2 days, indicating rapid disease progression (**Table 1**). The reported mean time from onset to colonic perforation in pediatric patients is 11 days and, thus, is longer in pediatric patients than in adult patients.^29)^

The identification of STEC O157 in stool samples is essential for the diagnosis of STEC infection and typically requires 3–5 days. Therefore, urgent surgery for STEC O157 infection may be performed before STEC O157 infection is definitively diagnosed, as was done in the present case. Except for the case reported by Nakagawa et al., in which the patient underwent surgery on day 35 of hospitalization, no patients have been preoperatively diagnosed with STEC O157 infection.^19)^ However, Shiga toxin was detected preoperatively in the case reported by Adachi et al., although a definitive diagnosis was not established.^28)^

## CONCLUSIONS

STEC O157 infection can rapidly progress to colonic necrosis requiring urgent surgery even in adults without HUS. Clinicians should be aware of this rare but potentially life-threatening complication.

## DECLARATIONS

### Availability of data and materials

Data are available on request to the corresponding author.
